# How could multimedia information about dental implant surgery effects patients’ anxiety level?

**DOI:** 10.4317/medoral.21254

**Published:** 2016-12-06

**Authors:** Hakki-Oguz Kazancioglu, Ameer-Shani Dahhan, Ahmet-Hüseyin Acar

**Affiliations:** 1 DDS, PhD. Department of Oral and Maxillofacial Surgery, Faculty of Dentistry, Bezmialem Vakif University, Istanbul, Turkey; 2 Private Practices, Istanbul, Turkey

## Abstract

**Background:**

To evaluate the effects of different patient education techniques on patients’ anxiety levels before and after dental implant surgery.

**Material and Methods:**

Sixty patients were randomized into three groups; each contained 20 patients; [group 1, basic information given verbally, with details of operation and recovery; group 2 (study group), basic information given verbally with details of operative procedures and recovery, and by watching a movie on single implant surgery]; and a control group [basic information given verbally “but it was devoid of the details of the operative procedures and recovery”]. Anxiety levels were assessed using the Spielberger’s State-Trait Anxiety Inventory (STAI) and Modified Dental Anxiety Scale (MDAS). Pain was assessed with a visual analog scale (VAS).

**Results:**

The most significant changes were observed in the movie group (*P* < 0.05). Patients who were more anxious also used more analgesic medication. Linear regression analysis showed that female patients had higher levels of anxiety (*P* < 0.05).

**Conclusions:**

Preoperative multimedia information increases anxiety level.

**Key words:**Implant, anxiety, pain, dental, video and patient knowledge.

## Introduction

Anxiety is an emotional reaction defined as stress, apprehension, nervousness and concerns caused by an intangible or diffuse advancing threat or approaching danger, accompanied by activation of the autonomous nervous system (1). Medical procedures almost always elicit a sense of loss of control, fear, helplessness, and feelings of stress and anxiety (2). Patients may not be able to cooperate with dentists when they experience anxiety during dental treatment, which may increase the amount of time needed and the level of difficulty of performing procedures, thereby causing unsatisfactory treatment results. Previous studies have found that individuals with a high fear of dentistry visited the dentist less often and had more decayed and missing teeth (1,2).

Dental implant is called a root form that replaces a natural tooth which is usually made from titanium and this treatment option has become widely applied globally, and acknowledged as the general dental treatment procedure for the cases with tooth loss. Dental implant application is a relatively simple surgical procedure for the surgeon; however, it is usually associated with a high level of anxiety and discomfort for the patient. Even hearing the words “implant surgery” increases the level of anxiety for many patients (3).

Explaining or facing the complications of operative procedures may affect the anxiety level of the patients. As an effective method for delivering knowledge to dental implant patients, written information has been used. However, these information sheets are not literate enough to read and understand by the patients (4). Some studies (5,6) have demonstrated that informing patients by means of videos, showing the operative procedures the patients will be subjected to, has been shown to decrease preoperative anxiety level and increase patient comprehension, although other studies have shown that these effects were relatively small (7) or increase anxiety level (8). The aim of the current study was to evaluate the effects of different patient education techniques on patients’ anxiety levels before and after dental implant surgery.

## Material and Methods

- Patients 

Patients were referred for dental implant treatment without distinction as to race, gender, or socioeconomic status. Each patient was asked whether he or she would like to participate after an explanation of the purpose of the study was given.

The patients who recently had radiotherapy in the maxillofacial area and received chemotherapy, had bisphosphonate usage and alcohol and drug addiction, excessive smokers, uncontrolled diabetes, rheumatoid arthritis and serious psychiatric and mental disorders were excluded from the study. All patients were informed about the dental implant surgery operation and possible complications. Informed consent was obtained from each participant. The study was designed according to the Declaration of Helsinki’s medical protocol and ethical permission was obtained from Bezmialem Vakif University Ethical Committee (No: 19/8, Date: 05/11/2014). As negative experiences about the procedure could also cause higher anxiety levels, patients with previously bad dental treatment history were also excluded from the study.

- Study Groups

Sixty patients were randomized into three groups; each contained 20 patients; two study groups [group 1, basic information given verbally, with details of operation and recovery stages; group 2 (study group), basic information, with details of operative procedures and recovery, given verbally and by watching a movie on single implant surgery]; and a control group [basic information given verbally “but it was devoid of the details of the operative procedures and recovery”]. The surgeon and patients were unaware of group allocations and baseline anxiety scoring. Group 1 and 2 patients were informed that local anesthesia would numb the operation area and they should not expect to feel pain. In addition, the surgeon would stop the procedure and provide further anesthesia if they felt any pain.

- Dental implant surgery video

The video, which we chose for this study, explained how a single dental implant surgery procedure is completed. The video was about two minutes long, without any emanations from the incision until suture. The movie began with an interview of the actual patient before surgery. The discussion included a description of the surgical procedure and the risks. The next scene showed the patient being moved into the operating theater and the surgeons performing the implant surgery.

- Dental implant surgery 

During this study, two types of dental implant systems from different countries (Bredent GmbH & Co.KG, Senden, Germany; Biohorizons(®) Implant Systems Inc., Birmingham, AL, USA) were used. All implants were made from titanium which is biocompatible for patients. One or two implants were placed in the mandible under local anesthesia by a single surgeon according to implant system recommendation.

- Measurement of anxiety and pain 

Anxiety levels were assessed using the Spielberger’s State-Trait Anxiety Inventory (STAI) and Modified Dental Anxiety Scale (MDAS). The STAI has 40 items, 20 items allocated to each of the S-Anxiety and T-Anxiety subscales (9). The State Anxiety Scale (STAI-S) evaluates the current state of anxiety, and the Trait Anxiety Scale (STAI-T) evaluates relatively stable aspects of “anxiety proneness,” including general states of calmness, confidence, and security (10). Modified dental anxiety scale (MDAS) includes a question about a local anesthetic injection. Each question has five scores ranging from ‘not anxious’, to ‘extremely anxious’, in an ascending order from 1 to 5. In this study, a Turkish translation of MDAS was used (11).

Pain was assessed with a visual analog scale (VAS). Statistical analysis was performed using SPSS 16.0.

## Results

Sixty (33 females and 27 males; mean age 44.63±8.22 years) patients fulfilled the inclusion criteria and consented to participate. The groups were similar in terms of gender distribution, age, and surgery time (*P* > 0.05). Demographic information that might affect anxiety and pain perception are shown in  Table 1. There was no significant difference in the mean surgery time (starting from the first incision to the last suture) between the groups (*p* > 0.05).

**Table 1:**
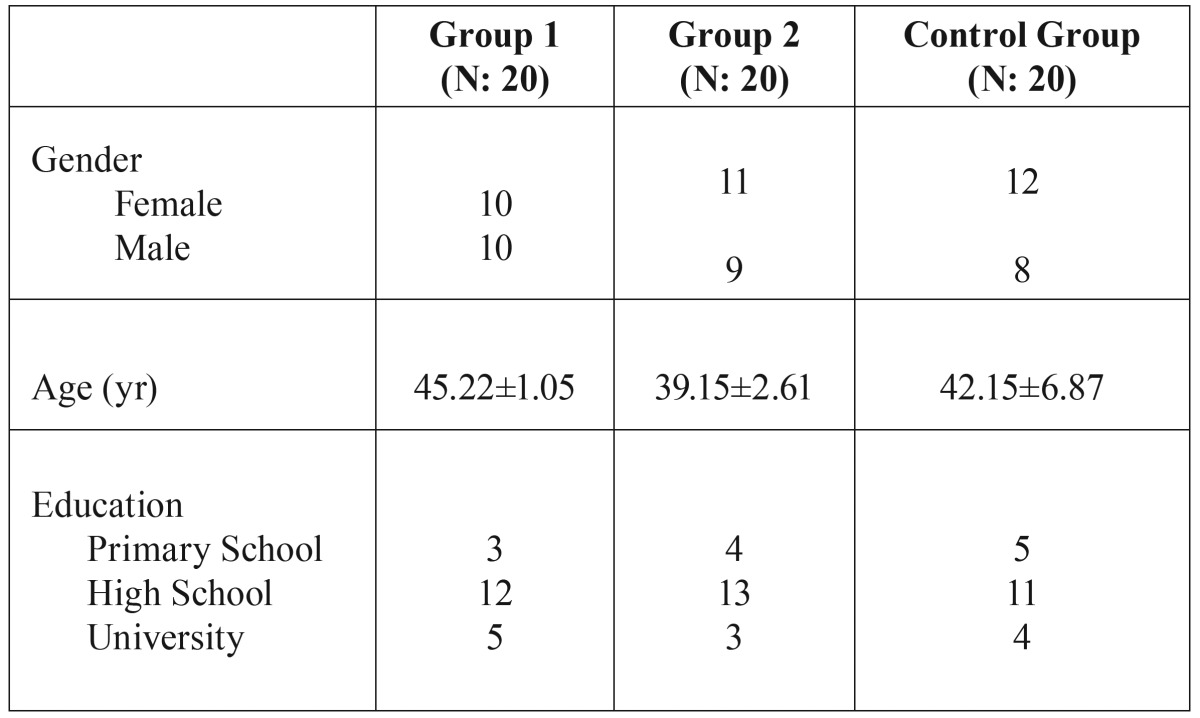
Patients’ characteristics (n = 60).

**Figure 1:**
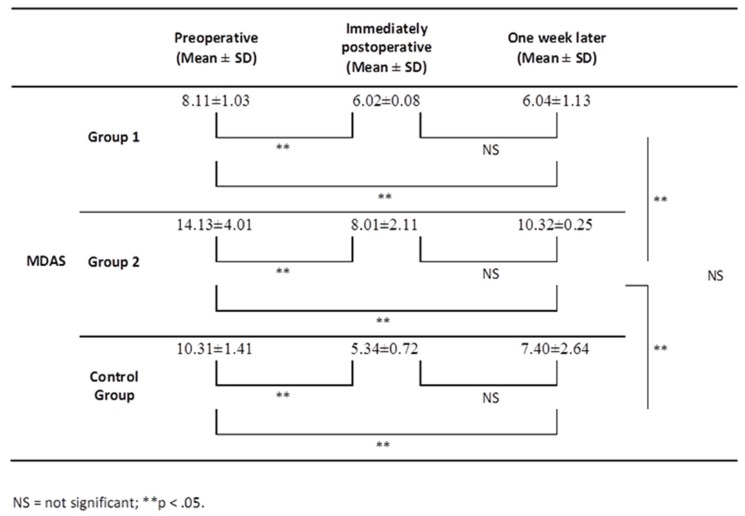
Mean MDAS scores at various points (n = 60).

MDAS scores showed that immediately postoperative and one week later the surgery was significantly lower than the scores before the surgical procedure in all groups (*p* < 0.05). When the groups were compared according to MDAS scores, movie group patients’ significantly higher MDAS scores compared the Group 1 and control group patients (*p* < 0.05) (Fig. 1).

**Figure 2:**
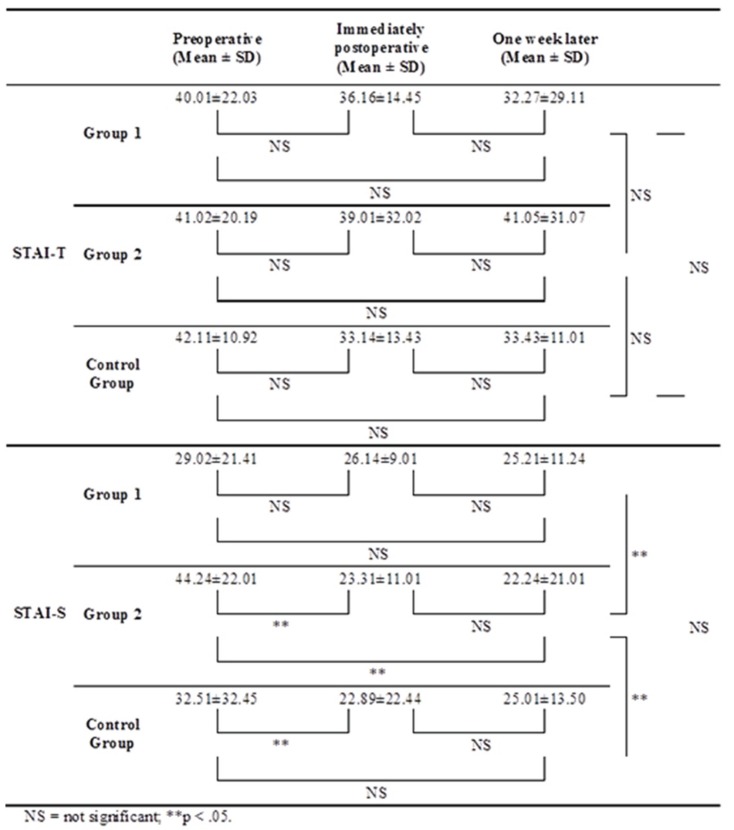
Mean STAI-T and STAI-S scores at various points (n = 60).

The frequency and percentage of the STAI-T and STAI-S scores are shown in figure 2. No differences in STAI-T scores were found among the groups (*p* > 0.05). In Group 2, the STAI-S scores immediately and one week after the surgical procedure were significantly lower than the scores before the surgical procedure (*P* < 0.05). In control group, immediately after the surgical procedure STAI-S scores were significantly higher than preoperative scores (*p* < 0.05). The most significant changes were observed in the movie group. This group’s patients were significantly more anxious before the dental implant surgery compared with those in group 1 and the control group (*p* < 0.05). Immediately after the surgical procedure, there was a significant decrease in anxiety compared with baseline in all groups; the patients in the movie group again displayed more anxiety compared with those in group 1 and the control group. Although the scores reported one week after the surgical procedure were slightly higher than those reported immediately after the surgical procedure, the difference was not significant.

Linear regression analysis showed that age, surgery time, and education level had no correlation with anxiety or pain levels; however, female patients had higher levels of anxiety (*P* < 0.05) ( Table 2).

**Table 2:**
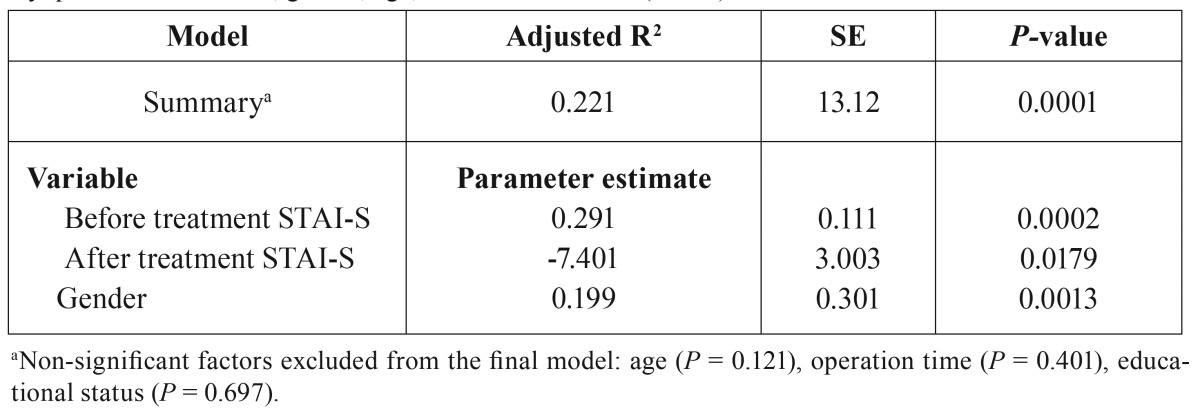
Linear multiple regression model of post-treatment STAI-S scores, adjusted for pretreatment anxiety questionnaire scores, gender, age, and education level (n = 60).

 Table 3 shows that patients with high anxiety had higher pain scores on visual analog scale (*P* < 0.001). Patients who were more anxious also used more analgesic medication.

**Table 3:**
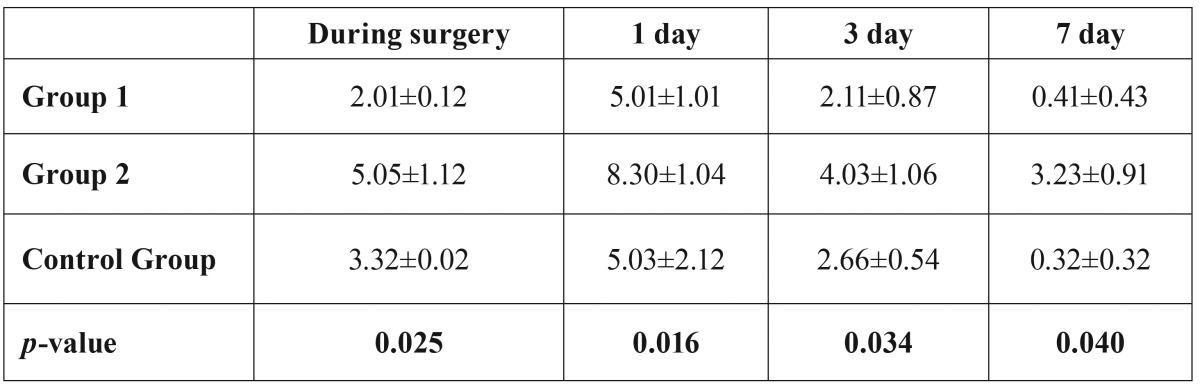
Mean ±SD pain scores on VAS (n = 60).

## Discussion

Finding information for medical treatments is quite easily available on the Internet. In our clinical experience, patients who had watched movies of dental implant surgery on the Internet were more anxious during the surgical procedure. However, no study has investigated the effect of watching live taped movies on patients’ anxiety levels before and after dental implant surgery. Therefore, the current study was designed to assess the level of anxiety and pain related to watching a preoperative video for the patients who want to see the procedure before the operation.

As many patients are afraid of dental treatments, it is not surprising to find most of the participants of the study having high STAI-T scores, which is consistent with the results of other studies (12,13). Dental implant surgery is a relatively simple procedure for the dentist; however, it is a complex procedure for the patients. Therefore, most of the implant patients have a high-state of anxiety prior to undergoing implant surgery.

The current study was to evaluate the profile of the patients and it was found that females have a higher anxiety level than males. These findings confirmed similar findings of previous clinical studies that showed higher levels of anxiety among females (14,15). However, some studies have not found any differences between the genders (16,17). The reason may be related to the suggestion that females are more likely to express their feelings and emotions than males. In addition, it could be attributed to the fact that men refuse to report symptoms they consider weak or unmasculine and tend to silently cope with anxiety.

The main finding of the current study is that patients who received information about the operative procedures and postoperative recovery, anxiety reduction would outweigh the fear provoked by the procedural details. However, watching a movie about surgery is a stressful event for patients. This finding is in accordance with previous findings that distressing experiences are likely to make patients sensitive and increase the risk of developing psychological distress following a later traumatic event (18). In 2014, Kazancioglu *et al.* (8) reported that preoperative multimedia information increases the level of STAI-S anxiety of wisdom teeth surgery patients. In addition, they found that providing information about the operative procedure and recovery helped improve the patients’ knowledge of the operation and might have reduced their uncertainty about the procedure. On the contrary, Kesari *et al.* (19) compared the anxiety levels of patients who watched their own cystoscopy and those who did not, however; they found no significant effect between groups. Tanaka *et al.* (20) reported high postoperative patient satisfaction after presenting live video on a monitor during arthroscopy. In the present study, watching a movie about implant surgery caused a significant increase in patient STAI-S scores compared with two groups.

The pain and anxiety relationship has been researched in many articles (21,22). Vallerand *et al.* (23) emphasises that increasing the quantity of preparatory information about the postoperative period significantly increases pain relief with the result of satisfaction with pain control without higher analgesic consumption. Information about the surgical procedure was intended to help patients obtain attentive, early interventions for controlling pain (24). Positive correlations were found between anxiety level and postoperative pain. However, as the articles depend on survey research, this subject is still debatable and future research should be considered using standardized procedures. The results of the present study demonstrated that the perception of pain related to implant surgery may have been influenced positively by written information provided preoperatively and that pain relief and satisfaction with pain control increased without an increase in the consumption of analgesics.

There are studies that aim to decrease anxiety level via different techniques (8,25). Thoma *et al.* (25) investigated the effectiveness of listening to music as an intervention in managing anxiety during dental treatments by integrating results from 16 different studies. They found the difference of effect was statistically significant. For instance, decreasing anxiety can reduce the amount of discomfort and pain experienced by patients, leading to lower dosages of required analgesics. In our study, patients who were more anxious also needed more analgesic medication.

In dental practice, it is difficult to determine the anxiety level in patients. Therefore, some anxiety scales may be used to assess anxiety levels. However, scoring and interpretation of the scale are based on self-report, and also patients who perceive themselves as being exposed to a potential risk factor may have higher scores. It must be acknowledged that exposure to risk factors was measured on the basis of self-report, rather than on the basis of an objective evaluation. In addition, some referrers may not give the correct or enough information about implant surgery, which may cause confusion and mistrust in the patient before the surgical procedure.

## Conclusions

Preoperative multimedia information of implant surgery increases the anxiety of patients undergoing implant surgery while written information given about operation stages and recovery with details decrease anxiety levels of the patients. In addition, the study results demonstrated that anxious patients need to use more analgesics for relief of their pain. Clinicians should be aware of the information they give to the patients and how this could change the patient’s anxiety and pain levels.
